# A Single Mutation of VP2 is Responsible for the Lethality and Antigenicity Differences between Novel Variant and Very Virulent IBDV Strains

**DOI:** 10.1155/2023/6684304

**Published:** 2023-11-14

**Authors:** Nan Jiang, Guodong Wang, Wenying Zhang, Yulong Wang, Xinxin Niu, Mengmeng Huang, Li Gao, Kai Li, Hongyu Cui, Changjun Liu, Yanping Zhang, Keyan Bao, Suyan Wang, Yuntong Chen, Xiaomei Wang, Yulong Gao, Xiaole Qi

**Affiliations:** ^1^Avian Immunosuppressive Diseases Division, State Key Laboratory for Animal Disease Control and Prevention, Harbin Veterinary Research Institute, Chinese Academy of Agricultural Sciences, No. 678 Haping Road, Xiangfang District, Harbin, Heilongjiang 150069, China; ^2^WOAH Reference Laboratory for Infectious Bursal Disease, Harbin Veterinary Research Institute, Chinese Academy of Agricultural Sciences, Harbin, China; ^3^National Laboratory of Biomacromolecules, CAS Center for Excellence in Biomacromolecules, Institute of Biophysics, Chinese Academy of Sciences, Beijing, China

## Abstract

Infectious bursal disease is an acute, immunosuppressive infectious disease in chickens caused by the infectious bursal disease virus (IBDV), which causes huge economic losses to the global poultry industry. Persistently circulating very virulent IBDV (vvIBDV) and newly emerging novel variant IBDV (nVarIBDV) are the two dominant epidemic strains of IBDV in East Asian countries such as China. Compared to lethal vvIBDV, nonlethal nVarIBDV has more insidious pathogenicity and can partially escape the immune protection of the existing vvIBDV vaccine, suggesting its potential adaptive survival strategy. However, the underlying molecular mechanism remains unknown. The viral capsid protein VP2 is closely related to cell tropism, virulence, and antigenic variation of IBDV. In this study, for the first time, we demonstrated that residue 279 of VP2 is responsible for the difference in pathogenicity between nVarIBDV and vvIBDV and that the D279N substitution reduces the lethality of vvIBDV from 70% to 0%. Moreover, a significant reduction in the viral load and inflammatory factor levels in the immune organs and blood of infected chickens may be important mechanisms for reducing the lethality of IBDV. Additionally, residue 279 was an important molecular basis for the antigenic differences between nVarIBDV and vvIBDV. D279N substitution reduced the neutralizing ability of vvIBDV antiserum against nVarIBDV by affecting the binding ability of antibodies and antigens. Our results indicate that nVarIBDV has an infection transmission strategy that facilitates its survival by hiding viral pathogenicity and escaping antiserum neutralization, which not only has significant implications for the systemic cognition of viral genetic evolution and pathogenesis but also provides new ideas for the comprehensive prevention and control of IBDV.

## 1. Introduction

Infectious bursal disease (IBD) is an acute, highly contagious, and immunosuppressive disease in chickens caused by the infectious bursal disease virus (IBDV) [1]. IBDV mainly damages chickens' central immune organ bursa, causing continuous immunosuppression and increasing their susceptibility to other pathogens [2]. IBDV is a nonenveloped, icosahedral, and double-stranded RNA virus belonging to the genus *Avibirnavirus* of the family *Birnaviridea* [1, 3]. Its genome contains two segments [4]. Segment A encodes the capsid protein VP2, structural protein VP3, protease VP4, and nonstructural protein VP5 [5, 6]. Hypervariable region (HVR) of VP2 (amino acids 206–350) is prone to mutation and has four loops (P_BC_, P_DE_, P_FG_, and P_HI_) on the outside [7], which are closely related to cell tropism [8–11], antigenic variation [12–14], and virulence of IBDV [9, 10]. Segment B encodes the VP1 protein with RNA-dependent RNA polymerase activity [15].

Classical IBDV was first discovered in Gumboro in the USA in 1957 [16]. Since then, it has undergone two large mutations, evolving as the variant IBDV (varIBDV) [17] and very virulent IBDV (vvIBDV) [18] in the late 1980s and early 1990s, respectively. The varIBDV can escape the protection of classical IBDV vaccine and is now predominantly epidemic in North America and Australia. The vvIBDV, with high lethality and transmission, induced huge losses to the global poultry industry in the past 30 years. The infection of vvIBDV is gradually being controlled via intense management and widespread use of vaccines. However, recently, novel variant strains of IBDV (nVarIBDV) [19, 20] have become widespread throughout East Asia [21]. Persistently circulating vvIBDV and newly emerging nVarIBDV are the two dominant epidemic strains of IBDV, at least in China [22, 23].

Compared to vvIBDV, nVarIBDV is not only more insidious in its pathogenicity (not lethal but still causes acute damage to central immune organs) but also able to escape the immune protection of the existing vvIBDV vaccines to a certain extent, indicating its strategy for adaptive survival. However, the exact underlying molecular mechanism remains unknown. This study is the first to identify residue 279 of VP2 as an important molecular basis for the difference in lethality between the two dominant IBDV strains and to reveal the molecular mechanism by which nVarIBDV persists in immunized flocks.

## 2. Materials and Methods

### 2.1. Viruses, Cell Cultures, Plasmids, and Antibodies

The nVarIBDV strain, SHG19 [19], and vvIBDV strain, HLJ0504 [24], were isolated and identified by the avian immunosuppressive disease division of the Harbin Veterinary Research Institute (HVRI), Chinese Academy of Agricultural Sciences (CAAS) (hereafter referred to as “our laboratory”). The rSHG19 and rHLJ0504 strains were rescued from the infectious clones of the two viruses using a reverse genetics system in our laboratory.

DF1 and 293T cells were cultured in the Dulbecco's modified Eagle's medium (Sigma-Aldrich, USA) supplemented with 10% fetal bovine serum (Sigma-Aldrich) at 37°C. DT40 cells were cultured in the Roswell Park Memorial Institute-1640 medium (Sigma-Aldrich) at 37°C supplemented with 10% fetal bovine serum (Sigma-Aldrich), 2% chicken serum (Sigma-Aldrich), 50 *μ*M *β*-mercaptoethanol (Gibco, USA), and 1% l-glutamine (Gibco).

Infectious clones of vvIBDV strain HLJ0504, pCAggsHLJ0504AHRT (pCAHLJA) and pCAggsHLJ0504BHRT (pCAHLJB), and nVarIBDV strain SHG19, pCAggsSHG19AHRT (pCASHGA) and pCAggsSHG19BHRT (pCASHGB), were constructed in our laboratory. Recombinant eukaryotic expression plasmids, pCAggsHLJ0504VP2 (pCAHLJVP2) expressing HLJ0504 VP2 and pCAggsSHG19VP2 (pCASHGVP2) expressing SHG19 VP2, were also constructed in our laboratory.

IBDV VP2 monoclonal antibody (MAb) 7D4 was produced and stocked in our laboratory. Goat antimouse IgG-FITC (F9006), rabbit antimouse IgG-TRITC (T2402), and rabbit antichicken IgY-FITC (F8888) antibodies were purchased from Sigma-Aldrich.

### 2.2. Animals

Specific pathogen-free (SPF) chickens were purchased from the National Poultry Experimental Animal Resource Bank of HVRI (CAAS) and raised in a negative-pressure isolator at the Experimental Animal Center.

### 2.3. Sequence Alignment of the VP2 HVR in IBDV

We compared the amino acid differences in the VP2 HVR between the nVarIBDV and vvIBDV representative strains (Table 1).

### 2.4. Construction of Point Mutated Infectious Clones of IBDV

To study the effect of residue 279 of VP2 on the pathogenicity of IBDV, single mutation D279N was introduced into the VP2 of segment A of pCAHLJA using primers pCA-EcoR1-F/HLJVP2-D279N-R and HLJVP2-D279N-F/pCA-Bgl2-R via polymerase chain reaction (PCR) for site-directed mutagenesis [25], and the mutated plasmid was named pCAHLJAD279N. Similarly, a single mutation, N279D, was introduced into the VP2 of segment A of pCASHG19 using primers pCA-EcoR1-F/SHGVP2-N279D-R and SHGVP2-N279D-F/pCA-Bgl2-R, and the mutated plasmid was named pCASHGAN279D. All primers used in this study are listed in Table 2.

### 2.5. Rescue of Point Mutation Viruses

With the reverse genetics system established in our laboratory [25, 26], pCAHLJAD279N/pCAHLJB and pCASHGAN279D/pCASHGB purified plasmids were used to rescue the point mutation viruses. Rescued viruses in the bursae were identified using reverse transcription (RT)-PCR, sequencing, and electron microscopy. All primers used for RT-PCR are listed in Table 2. Indirect immunofluorescence assay (IFA) was also performed for infected DT40 cells.

### 2.6. Viral Titration

As previously described, IBDV titers were determined using real-time quantitative PCR (RT-qPCR) [9]. Specific primers and TaqMan probes for chicken 28s rRNA and IBDV load for RT-qPCR are listed in Table 2. The cDNA copies were normalized to the 28s cDNA copies measured from the same samples. In addition, viral titers were determined using 50% tissue culture infective dose (TCID_50_) in DT40 cells. As these viruses did not exert any cytopathic effects on DT40 cells, an IFA mediated by an IBDV monoclonal antibody was used to determine TCID_50_.

### 2.7. Pathogenicity Assessment

Animal experiments were performed to evaluate the lethal characteristics of the point-mutant viruses. Three-week-old SPF chickens were divided into five groups of 10 chickens each: rHLJ0504 infection group, rHLJ-D279N infection group, rSHG19 infection group, rSHG-N279D infection group, and mock group. The infection dose was 2.5 × 10^5^ viral RNA copies/200 *μ*l per chicken via ocular and intranasal routes. The mock group was inoculated with equal phosphate-buffered saline (PBS; pH = 7.0). Clinical symptoms were observed using the mean symptomatic index (MSI) over 7 days. MSI ranged from 0 to 3 with increasing severity as previously described [27]. Survival curves were constructed for each group.

The second animal experiment was performed to understand further the pathogenicity and possible causes of point-mutant viruses in detail. Three-week-old SPF chickens were divided into five groups of 21 chickens each: rHLJ0504 infection group, rHLJ-D279N infection group, rSHG19 infection group, rSHG-N279D infection group, and mock group. Chickens received 200 *μ*l virus (1 × 10^2^ viral RNA copies) or PBS (pH = 7.0) per chicken. Three chickens in each group were randomly euthanized each day, anticoagulated blood and serum were collected, organ lesions were observed, the bursa, spleen, and thymus were collected, and the organ weight ratio was calculated as follows: Organ weight ratio = (organ weight (g) × 1,000)/body weight (g). In addition, parts of the organs were used for viral load detection. The bursa, spleen, and thymus tissues were fixed in 10% formalin and stained with hematoxylin and eosin for histopathological examination.

### 2.8. Measurement of Interleukin (IL)-1*β* Levels

RT-qPCR was used for relative quantifying inflammatory cytokine IL-1*β* expression in tissues based on the 2^−*ΔΔCt*^ method. Primers used for IL-1*β* and *β*-actin are listed in Table 2. According to the manufacturer's instructions, IL-1*β* levels in serum samples were measured using enzyme-linked immunosorbent assay (ELISA) kits (Cloud-Clone Corp., China).

### 2.9. Antiserum Neutralization Assays

To evaluate the influence of residue 279 mutation on the antigenicity of IBDV, the neutralization ability of HLJ0504 antiserum against the homologous virus rHLJ0504, mutated virus rHLJ-D279N, and heterologous virus rSHG19 was determined. The neutralization ability of SHG19 antiserum against the homologous virus rSHG19, mutated virus rSHG-N279D, and heterologous virus rHLJ0504 was also determined. As previously described, neutralization assays were performed on DT40 cells using IFA [26].

### 2.10. Measurement of Antigen–Antibody Affinity

HLJ0504 antiserum-mediated IFA was performed to detect the effect of residue 279 of VP2 on the antigen–antibody affinity. The single-point mutation D279N was introduced in VP2 of HLJ0504 strain using primers PCA-HLJVP2-F/HLJVP2-D279N-R and HLJVP2-D279N-F/PCA-HLJVP2-R (Table 2), and the recombinant mutated plasmid was named pCAHLJVP2-D279N. The pCAHLJVP2, pCAHLJVP2-D279N, and pCASHGVP2 plasmids were transfected into 293T cells to express homologous VP2 protein (HLJ-VP2), the VP2 with D279N mutation (HLJ-VP2-D279N), and heterologous VP2 (SHG-VP2). At 24 hr post-infection (hpi), HLJ0504 antiserum-mediated IFA was performed. First, different dilutions (1 : 100, 1 : 500, and 1 : 2,500) of HLJ0504 antiserum were added into 293T cells expressing different VP2 proteins, and the cells were incubated at 37°C for 1 hr. Then, the antiserum was discarded, cells were washed three times with PBS, IBDV VP2 Mab 7D4 was added, and incubated at 37°C for 1 hr. Cells were washed three times with PBS, added anti-chicken FITC, and incubated for 45 min to detect HLJ0504 antiserum. Cells were washed three times with PBS and cultured with anti-rat TRITC for 45 min to detect viral VP2 protein expression. Finally, cells were washed three times with PBS and observed using an inverted fluorescence microscope. Image J software was used for visual, quantitative detection, and analysis of fluorescence signals, and the percentage of HLJ0504 antiserum-binding positive cells (green fluorescence)/different VP2 protein expression cells (red fluorescence) was calculated. As the epitope targeted by VP2 MAb 7D4 is conserved in various IBDV strains, there was no difference in its affinity for different VP2. If the HLJ0504 antiserum has a different affinity for different VP2 proteins, it will be reflected by this percentage.

### 2.11. Structural Comparisons of VP2 of IBDV Strains with Differences in Residue 279

Three-dimensional (3D) structures of the VP2 proteins of the SHG19 and HLJ0504 strains were predicted using the I-TASSER algorithm and subsequently analyzed and compared using the PyMOL software 2.3.

### 2.12. Statistical Analysis

All data were analyzed via unpaired *t*-tests using the Prism software 9.0 (GraphPad Software, Inc.). Statistical significance was set at.

## 3. Results

### 3.1. Influence of Residue 279 Mutation of VP2 on the Pathogenicity of IBDV

**Figure 1:**
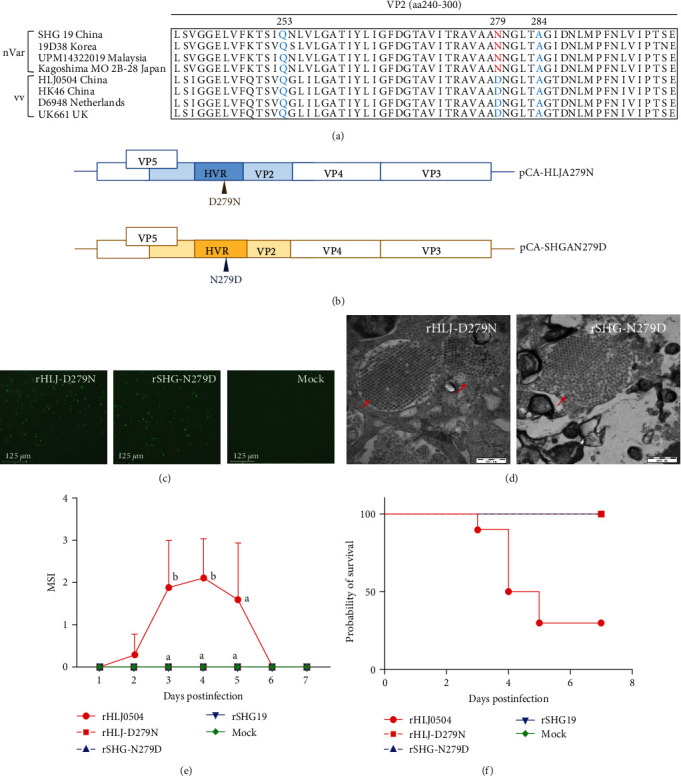
Influence of residue 279 of VP2 on the pathogenicity difference between novel variant infectious bursal disease virus (nVarIBDV) and very virulent IBDV (vvIBDV). (a) Amino acid differences between nVarIBDVs (SHG19, 19D38, UPM14322019, and Kagoshima MO 2B-28 strains) and vvIBDVs (HLJ0504, HK46, D6948, and UK661 strains) in the hypervariable region of VP2. nVar, nVarIBDV; vv, vvIBDV. (b) Schematic diagram of the infectious clones of segment A with residue 279 mutation based on the backbone of HLJ0504 or SHG19 strain. (c) Immunofluorescence assay (IFA) of point mutation viruses rHLJ-D279N and rSHG-N279D in DT40 cells with MAb against IBDV VP2 at 24 hr postinfection (hpi). (d) Electron microscopy detection of point mutation viruses rHLJ-D279N and rSHG-N279D in the bursa. The viruses arranged in crystal lattices were marked with arrows. (e) Mean symptomatic index (MSI) of chickens infected with rHLJ0504, rHLJ-D279N, rSHG-N279D, or rSHG19, with a dose of 2.5 × 10^5^ viral RNA copies/200 *μ*l. Treatments with different lowercase letters differ significantly at their confidence level (*P* < 0.05). (f) Survival curve of the infected chickens within the observation period of 7 days.

Differences in the amino acids in the HVR of HLJ0504 and SHG19 VP2 were compared (Figure 1(a)). Residues 253, 279, and 284 of VP2 have been reported as being deeply involved in the virulence of IBDV [10, 11], and residues 253 and 284 have been identified as the determined virulence-determining sites for various strains [9]. HLJ0504 and SHG19 were identical at residues 253 and 284; therefore, it was speculated that residue 279 was the critical amino acid that influenced the difference in pathogenicity. D279N was introduced into VP2 of the HLJ0504 strain to verify this speculation, while N279D was introduced into the SHG19 strain (Figure 1(b)). The corresponding mutated viruses, rHLJ-D279N and rSHG-N279D were rescued. The IFA results showed that DT40 cells infected with rHLJ-D279N and rSHG-N279D showed a positive green fluorescence signal at 24 hpi (Figure 1(c)). Electron microscopy showed a lattice-like regular arrangement of nonenveloped virions with a diameter of ∼60 nm in the bursa of the infected chickens (Figure 1(d)). RT-PCR and sequencing further confirmed the successful rescue of these mutated viruses.

The first animal experiment involving infection with mutated viruses was performed to evaluate the effect of residue 279 on IBDV pathogenicity. MSI showed that the rHLJ0504 group showed severe clinical symptoms, starting from 2 days post-infection (dpi), peaking at 3–5 dpi, and disappearing at 6 dpi. The rHLJ-D279N, rSHG-N279D, and rSHG19 groups exhibited no clinical symptoms (Figure 1(e)). Survival curves showed that chickens in rHLJ0504 groups 1, 4, and 2 died at 3, 4, and 5 dpi, respectively. There was no mortality from 6 dpi, and the cumulative mortality rate was 70% (7/10). No mortality was observed in the rHLJ-D279N, rSHG-N279D, and rSHG19 groups (Figure 1(f)). These data show that residue 279 of VP2 contributes to the difference in lethality and morbidity between nVarIBDV and vvIBDV.

### 3.2. Influence of Residue 279 Mutation of VP2 on Immune Organs Damage by IBDV

In the second animal experiment, we further investigated the influence of residue 279 on lesions of the main immune organs caused by IBDV.

#### 3.2.1. Bursa

**Figure 2:**
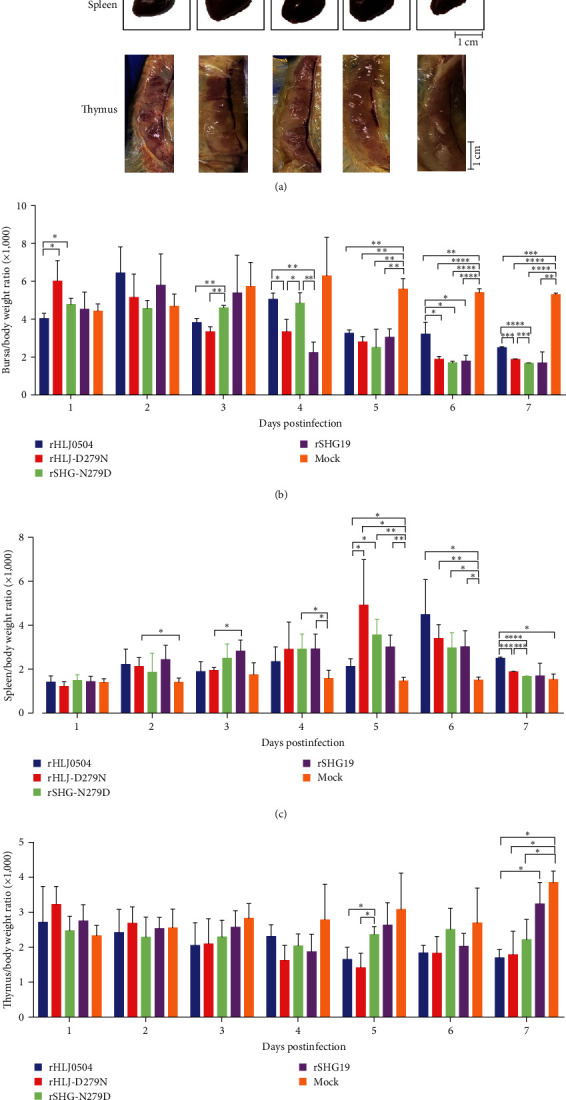
Tissue lesions in major immune organs induced by different mutated IBDVs. (a) Clinical necropsy of the bursa, spleen, and thymus tissues of chickens infected with rHLJ0504, rHLJ-D279N, rSHG-N279D, or rSHG19 at 5 dpi. (b) Bursa/body weight. (c) Spleen/body weight. (d) Thymus/body weight. Error bars represent the standard deviation (SD).  ^*∗*^*P* < 0.05;  ^*∗∗*^*P* < 0.01;  ^*∗∗∗*^*P* < 0.001;  ^*∗∗∗∗*^*P* < 0.0001.

**Figure 3:**
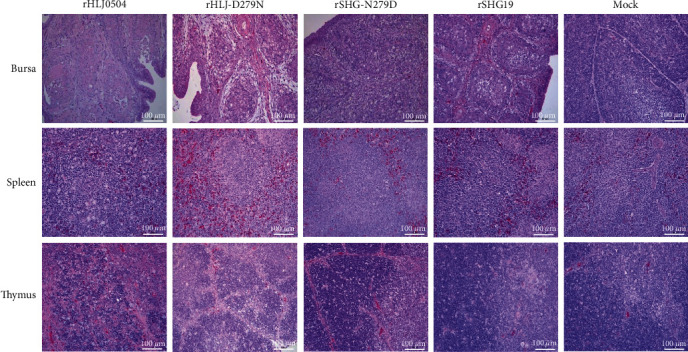
Histopathological examination of the bursa, spleen, and thymus tissues of chickens infected with rHLJ0504, rHLJ-D279N, rSHG-N279D, or rSHG19 at 5 dpi.

The clinical autopsy showed that the rHLJ0504 group exhibited bursal lesions (atrophy, yellowing, and inflammatory mucus exudation) from 2 dpi, with severe hemorrhage seen in individual bursa at 3–5 dpi. The bursa in the rHLJ-D279N, rSHG19, and rSHG-N279D infection groups also showed typical lesions from 2 dpi but no severe hemorrhage. Bursae at 5 dpi are shown in Figure 2(a). Analysis of the bursa/body weight ratio (Figure 2(b)) showed that the mean ratio of all four infected groups was lower than that of the mock control group from 3 dpi, and the difference was significant from 5 dpi to the mock control group. Histopathological examination revealed that all four IBDV strains induced severe bursal damage. From 2 dpi, necrosis and reduction of lymphocytes in the bursa were observed in all infected groups. Many lymphocyte disintegrations, necrosis, and follicular atrophy were observed in the bursa at 3–7 dpi. Pathological sections of the bursa at 5 dpi are shown in Figure 3. The combined results showed that all four strains of IBDV caused serious damage to the bursa, lymphocyte disintegration, and necrosis, and rHLJ0504 caused even more serious hemorrhage in the bursa.

#### 3.2.2. Spleen

The clinical autopsy showed that the spleens of all four infection groups were swollen from 2 dpi compared to the mock group. The spleens at 5 dpi are shown in Figure 2(a). The spleen/body ratios (Figure 2(c)) confirmed splenomegaly in all four infection groups from 2 dpi. Compared with the rHLJ0504 group, the rHLJ-D279N group had lower spleen/body ratios at 6 and 7 dpi, with a significant difference at 7 dpi. Histopathological examination showed that the rHLJ0504 group had an accumulation of red marrow erythrocytes and a decrease in white marrow lymphocytes from 2 dpi, a significant decrease in white marrow lymphocytes, and a proliferation of macrophages from 3 to 6 dpi, an alleviation in pathological damage, and a slight decrease in white marrow lymphocytes at 7 dpi. The remaining infection groups showed no lesions in the spleen at 1–7 dpi. Pathological sections of the spleen at 5 dpi are shown in Figure 3. The combined results showed that the four viruses caused a certain percentage of splenomegaly at 2 dpi. The rHLJ0504 caused an extensive reduction in the number of lymphocytes and proliferation of macrophages in the spleen, whereas rHLJ-D279N did not.

#### 3.2.3. Thymus

The clinical autopsy results showed that compared to the mock group, individual chickens in the rHLJ0504 and rHLJ-D279N infection groups presented with thymus atrophy from 3 dpi, and the thymus in the rHLJ0504 group was accompanied by hemorrhagic spots. Compared with the rSHG19 group with no visible lesions, the rSHG-N279D infection group showed individual thymus atrophy from 3 dpi. The thymus at 5 dpi is shown in Figure 2(a). Thymus/body weight ratios (Figure 2(d)) further confirmed these findings. Histopathological examination revealed that the thymuses in the rHLJ0504 infection group showed lymphocytopenia and macrophage hyperplasia from 2 dpi, massive lymphocytopenia, and significant macrophage hyperplasia from 3 to 7 dpi, and peripheral lobular hemorrhage from 3 to 5 dpi. Comparatively, the thymuses in the rHLJ-D279N group showed mild lymphocytopenia and mild macrophage hyperplasia at 3–5 dpi, and the lesions disappeared at 6 and 7 dpi. The thymus in the rSHG19 and rSHG-N279D infection groups showed a slight lymphocyte reduction at 4–7 dpi. The pathological sections of the thymus at 5 dpi are shown in Figure 3. The combined results indicated that rHLJ0504 and rHLJ-D279N caused a certain proportion of thymic atrophy and reduced cortical lymphocyte necrosis, with milder lesions in the rHLJ-D279N group. No atrophy was observed in the rSHG19 group, and rSHG-N279D caused a certain proportion of thymic atrophy.

Comprehensive results of clinical autopsy, organ/weight ratio, and pathological examination showed that rHLJ0504 caused the most severe damage to the immune organs of the bursa, spleen, and thymus, whereas D279N mutation significantly reduced the degree of damage to these immune organs caused by rHLJ0504. The rSHG19 also caused severe damage to the bursa but no significant damage to the spleen and thymus, whereas the N279D mutation enhanced the damage to the thymus caused by rSHG19 to some extent.

### 3.3. Influence of Residue 279 Mutation of VP2 on IBDV Tissue Distribution and Replication in Vivo

**Figure 4:**
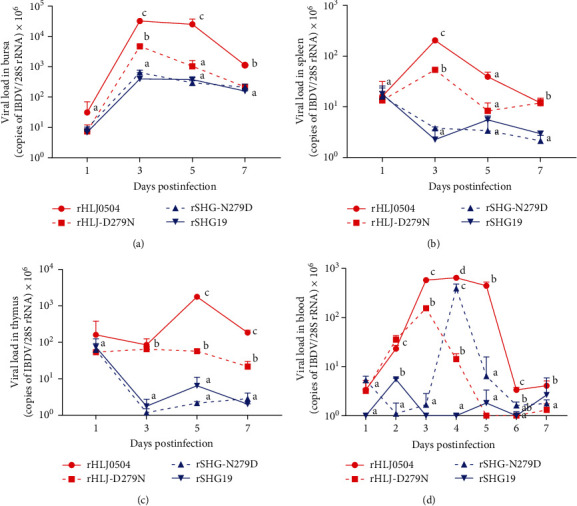
Viral loads of IBDV in the major immune organs and blood samples of chickens infected with rHLJ0504, rHLJ-D279N, rSHG-N279D, or rSHG19 at 1, 3, 5, and 7 dpi, respectively. (a) Bursa. (b) Spleen. (c) Thymus. (d) Blood. Error bars represent the SD. Treatments with different lowercase letters differ significantly at their confidence level (*P* < 0.05).

This study further compared the influence of residue 279 mutations on tissue distribution and replication efficiency of IBDV in vivo. RT-qPCR results showed that each infection group had the highest viral load in the bursa and lower viral loads in the spleen and thymus. In the bursa (Figure 4(a)), rHLJ0504 had the highest viral load, with replication peak at 3–5 dpi. Comparatively, the replication peak of rHLJ-D279N occurred at 3 dpi, and its titer then decreased rapidly. Additionally, the viral load of rHLJ-D279N was lower than that of rHLJ0504 in the bursa. The viral load of rHLJ0504 in the bursa at 3, 5, and 7 dpi was 6.85, 24.31, and 5.22 times higher than that of rHLJ-D279N, respectively. There were no significant differences in virus titers in the bursa between the rSHG19 and rSHG-N279D groups. In the spleen (Figure 4(b)), rHLJ0504 also had the highest viral load, with a replication peak at 3 dpi.

In comparison, the viral load of rHLJ-D279N in the spleen was lower than that of rHLJ0504. The viral load of rHLJ0504 in the spleen at 3 and 5 dpi was 3.86 and 7.00 times higher than that of rHLJ-D279N, respectively. There was no significant difference in the viral titers in the spleen between the rSHG19 and rSHG-N279D groups. In the thymus (Figure 4(c)), rHLJ0504 had the highest viral load, with a replication peak at 5 dpi. Comparatively, the viral load of rHLJ-D279N was generally lower than that of rHLJ0504 in the thymus. The viral load of rHLJ0504 in the thymus at 5 and 7 dpi was 31.35 and 8.51 times higher than that of rHLJ-D279N, respectively. There was no significant difference in viral titers in the thymus between the rSHG19 and rSHG-N279D groups.

In the blood (Figure 4(d)), the viral titer of rHLJ0504 was significantly higher than that of the other groups, and the viral titer of rSHG19 was very low at all time points. rHLJ0504 presented a replication peak in the blood at 3–5 dpi. Comparatively, the replication peak of rHLJ-D279N was at 3 dpi, then rapidly decreased. Even at the replication peak at 3 dpi, the viral load of rHLJ0504 was 45.38 times higher than that of rHLJ-D279N. Compared with rSHG19, rSHG-N279D rapidly increased the viral load to 607.39 times higher than rSHG19 at 4 dpi and then rapidly decreased.

The lethal vvIBDV rHLJ0504 strain has the highest viral load in the bursa and blood, followed by the thymus and spleen; the virus load of the nonlethal nVarIBDV rSHG19 strain in the three lymphatic organs and blood was lower. The D279N mutation decreased the viral titer of rHLJ0504 in lymphoid organs, such as the bursa, spleen, thymus, and blood; the N279D mutation significantly increased the viral load of rSHG19 in the blood at 4 dpi, although it did not increase the viral titer in lymphatic organs.

### 3.4. Influence of Residue 279 Mutation of VP2 on IBDV-Induced Inflammatory Response

**Figure 5:**
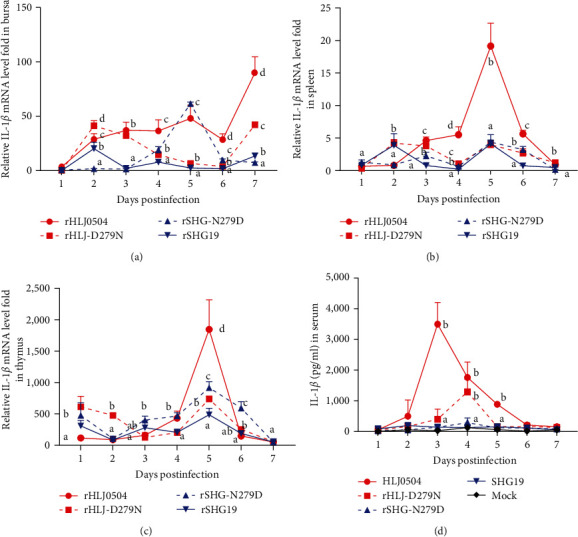
Interleukin (IL)-1*β* levels in the major immune organs and serum samples of chickens infected with rHLJ0504, rHLJ-D279N, rSHG-N279D, or rSHG19. (a) Bursa. (b) Spleen. (c) Thymus. (d) IL-1*β* levels in the serum samples were determined using an enzyme-linked immunosorbent assay (ELISA) kit (Cloud-Clone). Error bars represent the SD. Treatments sharing different lowercase letters differ significantly at their confidence level (*P* < 0.05).

The results of RT-qPCR for IL-1*β* in the bursa are shown in Figure 5(a). Overall, IL-1*β* was most markedly upregulated in the rHLJ0504 infection group within 7 dpi, whereas its upregulation was not significant in the rSHG19 infection group. In the rHLJ0504 group, IL-1*β* in the bursa increased gently from 1 to 5 dpi, declined moderately at 6 dpi, and increased rapidly to a maximum value at 7 dpi. IL-1*β* in the rHLJ0504 group at 4–7 dpi was 2.14, 7.50 times higher than in the rHLJ-D279N group. Compared with the rSHG19 group, IL-1*β* in the rSHG19-N279D group significantly increased at 5 dpi to 26.42 times that of the rSHG19 group and rapidly decreased. RT-qPCR results for IL-1*β* in the spleen are shown in Figure 5(b). Overall, IL-1*β* was most significantly upregulated in the rHLJ0504 infection group within 7 dpi, whereas the content of IL-1*β* was very low in the rSHG19 infection group. The induced IL-1*β* in the rHLJ0504 infection group peaked at 5 dpi and rapidly decreased. At 4–6 dpi, IL-1*β* mRNA levels in the rHLJ0504 infection group were 1.62–5.13 times higher than those in the rHLJ-D279N group. The rSHG-N279D group's IL-*β* at 3 and 6 dpi were 2.85 and 3.10 times higher than those of the rSHG19 group. RT-qPCR results for IL-1*β* in the thymus are shown in Figure 5(c). Overall, the mRNA levels of IL-1*β* peaked at 5 dpi in all four infection groups, with the IL-1*β* value in the rHLJ0504 group being the highest. At 4 and 5 dpi, IL-1*β* in the rHLJ0504 group were 2.15 and 2.50 times higher than those in the rHLJ-D279N. The IL-1*β* in the rSHG-N279D group were 1.43–3.15 times higher than those in the rSHG19 group at 3–6 dpi.

ELISA results for IL-1*β* in serum are shown in Figure 5(d). The rHLJ0504 group showed the highest IL-1*β* levels within 7 dpi among the four infection groups. IL-1*β* levels in serum of the rHLJ0504 group gradually increased at 1 and 2 dpi, reached a maximum value of 3,500 pg/ml at 3 dpi, and gradually decreased. Compared to rHLJ0504, the IL-1*β* peak of the rHLJ-D279N group was delayed from 3 to 4 dpi, and its maximum value (1,289 pg/ml) was ∼63% lower than that of the rHLJ0504 group. There was no significant difference in IL-1*β* levels in serum between the rSHG19 and rSHG19-N279D groups.

### 3.5. Influence of Residue 279 Mutation of VP2 on IBDV Escape from Antiserum Neutralization

**Figure 6:**
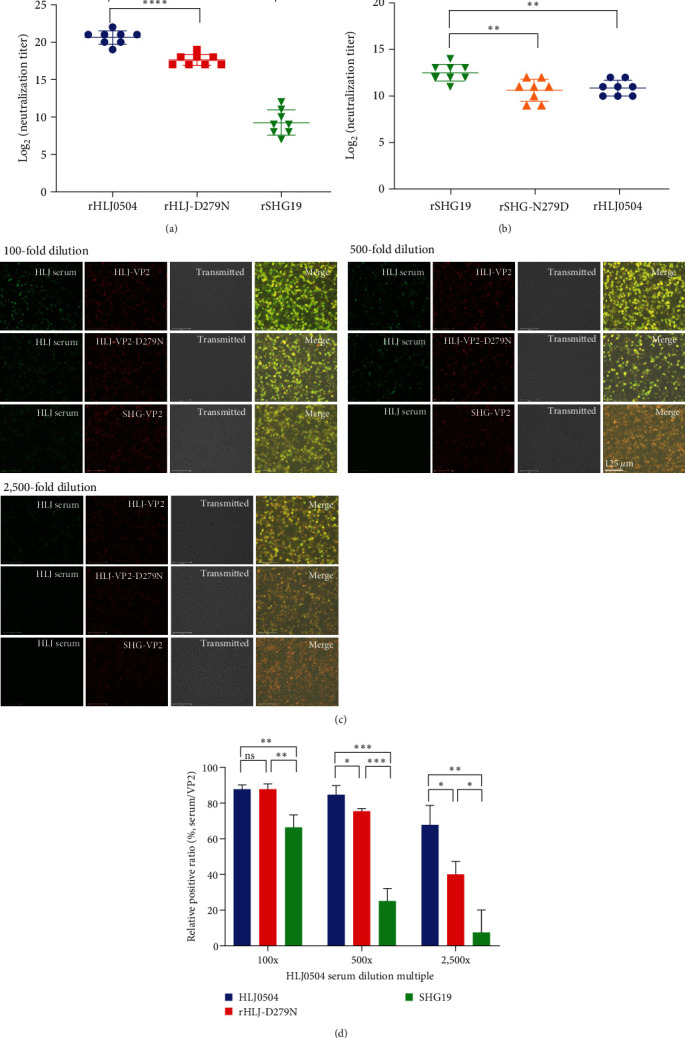
Influence of residue 279 mutation on antiserum neutralization of IBDVs. (a) Neutralizing test of HLJ0504 antiserum against the homologous virus rHLJ0504, point mutation virus rHLJ-D279N, and heterogenous virus SHG19. (b) Neutralizing test of SHG19 antiserum against the homologous virus rSHG19, point mutation virus rSHG-N279D, and heterogenous virus rHLJ0504. (c) Binding capacities of HLJ0504 antiserum to the homologous VP2 protein HLJ-VP2, point-mutant VP2 protein HLJVP2-D279N, and heterologous VP2 protein SHG-VP2 determined via IFA. Green indicates the HLJ0504 antiserum-binding positive cell. Red indicates different VP2 protein expression cells. (d) Percentage of HLJ0504 antiserum-binding positive cells (green fluorescence)/different VP2 protein expression cells (red fluorescence). Error bars represent the SD.  ^*∗*^*P* < 0.05;  ^*∗∗*^*P* < 0.01;  ^*∗∗∗*^*P* < 0.001;  ^*∗∗∗∗*^*P* < 0.0001.

To further determine whether the residue 279 mutation influenced the antigenicity of IBDV, antigenic differences between the mutant virus and its parental virus were detected. The neutralization titers of HLJ0504 antiserum against the homologous virus rHLJ0504, point-mutant virus rHLJ-D279N, and heterologous virus rSHG19 were 20.6 ± 0.9 log_2_, 17.6 ± 0.7 log_2_, and 9.3 ± 1.6 log_2_, respectively. The neutralizing titer of the HLJ0504 antiserum against rHLJ-D279N was reduced by 3 log_2_ compared to that against rHLJ0504 (Figure 6(a)). A neutralization test of the SHG19 antiserum against mutant viruses was also conducted. The neutralization titers of SHG19 antiserum against the homologous virus rSHG19, point-mutant virus rSHG-N279D, and heterologous virus rHLJ0504 were 12.3 ± 1.0 log_2_, 10.6 ± 1.1 log_2_, and 10.9 ± 0.8 log_2_, respectively. The neutralizing titer of the SHG19 antiserum against rSHG-N279D was reduced by 1.75 log_2_ compared to that against rSHG19 (Figure 6(b)). These data indicate that the D279N mutation significantly influences the antigenicity of rHLJ0504, whereas the N279D mutation significantly influences the antigenicity of rSHG19.

### 3.6. Influence of Residue 279 Mutation of VP2 on Antigen–Antibody Affinity

Differences in the affinity of the HLJ0504 antiserum for the homologous VP2 protein HLJ-VP2, the point mutation VP2 protein HLJ-VP2-D279N, and the heterologous VP2 protein SHG-VP2 were detected by IFA. The results showed (Figures 6(c) and 6(d)) that the binding ability of HLJ0504 antiserum to the VP2 protein was dose dependent. Compared to the ability of HLJ0504 antiserum to bind homologous HLJ-VP2, a significant decrease of 24% in its ability to bind heterologous SHG-VP2 occurred when HLJ0504 antiserum was diluted 100-fold. When the HLJ0504 antiserum was diluted 500- or 2,500-fold, its binding ability to SHG-VP2 decreased by 70% or 89%, suggesting a significant antigenic difference between HLJ-VP2 and SHG-VP2. In addition, compared to HLJ0504 antiserum binding to homologous HLJ-VP2, its binding capacity to HLJ-VP2-D279N showed a significant decrease of 11% when the HLJ0504 antiserum was diluted 500-fold. When the antiserum was diluted 2,500-fold, the binding capacity decreased by 41%, suggesting that the D279N mutation reduced the binding ability of HLJ0504 antiserum to HLJ-VP2. These data demonstrate that residue 279 of VP2 is an important amino acid responsible for the antigenicity difference between nVarIBDV and vvIBDV.

**Figure 7:**
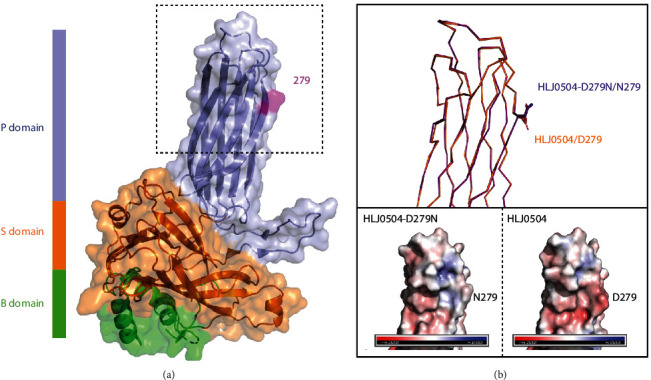
Structural comparisons of VP2 of different IBDV strains. (a) Structure of IBDV VP2. P, S, and B domains were marked with different colors. The key residue 279 was highlighted. (b) Top panel, zoomed-in view of the dashed box in (a) showing the comparison of VP2 between HLJ0504 (orange) and HLJ0504-D279N (purple) strains. Side chains of residue 279 were displayed as sticks. Bottom panel, same view of top panel showing the electrostatic surface representations of HLJ0504-D279N (left) and HLJ0504 (right) strains.

The predicted structure of VP2 showed that residue 279 was located in the outermost region of the P_FG_ loop of viral VP2 (Figure 7(a)). Compared with asparagine (D) at position 279 of VP2 of the HLJ0504 strain, asparagine (N) at position 279 of the mutant virus rHLJ-D279N strain exhibited different side chains. The D279N mutation significantly altered the surface potential of this region (Figure 7(b)), which may affect the binding affinity of virus antigens and antibodies as well as other biological characteristics of the virus.

## 4. Discussion

The emergence of strains with reduced pathogenicity is an important strategy for viral evolution. Previously, an epidemic of less virulent natural mutant viruses increased the difficulty of the early diagnosis of African swine fever and created new challenges for its control [28, 29]. Compared to the persistently circulating vvIBDV, the newly emerging nVarIBDV does not directly kill chickens and does not exhibit obvious clinical symptoms, resulting in this disease often being ignored. Moreover, nVarIBDV can partially evade the immune protection of the vvIBDV vaccine in immunized chicken flocks [30], indicating its survival strategy under strong immune prevention and control measures. The infection of nVarIBDV causes acute damage to immune organs and the disintegration and necrosis of B lymphocytes, weakening the immunity of chicken flocks and significantly affecting their production performance, such as weight gain [19], and immune effects of vaccines, such as avian influenza and Newcastle disease vaccines [21]. Chicken flocks are prone to great losses due to secondary or concurrent infections [20]. However, the specific molecular mechanisms underlying the significant differences in the pathogenicity of the two dominant epidemic strains, vvIBDV and nVarIBDV, remain unclear.

*VP2* is a virulence-determining gene in IBDV. Synergistic mutations at residues 253 and 284 of VP2 can attenuate vvIBDV to a nonlethal strain [9]. However, sequence analysis revealed that nVarIBDV and vvIBDV were identical at residues 253 and 284 of VP2, respectively. Residue 279 of VP2 has also been reported to be associated with viral virulence; however, reports on this are inconsistent for different IBDV strains [10, 11, 31]. The function of residue 279 of VP2 may vary depending on the genomic environment of the viral strain [31]. Residue 279 of VP2 differs in nVarIBDV and vvIBDV, indicating that this residue may contribute to the differences in pathogenicity between nVarIBDV and vvIBDV. In this study, for the first time, a single mutation in D279N was found to decrease the mortality of vvIBDV from 70% to 0%, suggesting that residue 279 of VP2 is an important amino acid influencing the difference in pathogenicity (especially the difference in lethality) between nVarIBDV and vvIBDV.

D279N was introduced into VP2 of the vvIBDV HLJ0504 strain to explore the possible mechanism further, whereas N279D was introduced into the nVarIBDV SHG19 strain. Differences in tissue distribution and replication in vivo between the mutated and parental viruses were detected. Among the immune organ and blood samples evaluated, lethal vvIBDV rHLJ0504 had the highest viral load in the bursa and blood, followed by the thymus and spleen. Notably, nonlethal nVarIBDV rSHG19 had a lower viral load in all three immune organs and blood. Clinical observations and histopathological examination revealed that rHLJ0504 caused the most severe damage to the immune organs of the bursa, spleen, and thymus. rSHG19 also severely damaged the bursa but not the spleen and thymus. vvIBDV rHLJ0504 replicated rapidly in the target organ of the bursa, causing acute damage to immune organs via blood transmission and may be an important cause of mortality. The data on mutated viruses revealed that the D279N substitution significantly lowered the viral replication of rHLJ0504 in the immune organs of the bursa, spleen, thymus, and blood. In contrast, the N279D substitution significantly increased the viral replication of rSHG19 in the blood at 4 dpi. However, it did not enhance the viral titer of rSHG19 in the immune organs, indicating that residue 279 of VP2 significantly influences the replication efficiency of IBDV in immune organs, such as the bursa, spleen, and thymus, and affects the viral load in the blood, subsequently causing damage to major immune organs and influencing the lethality of IBDV.

Animal experiments also revealed that inflammatory damage to immune organs varied among the pathogenic IBDV strain infection groups. Inflammatory response is an important immune defense mechanism in organisms. When a pathogen attacks an organism, it is in a state of self-protection and damage repair. IL-1*β* is a typical proinflammatory cytokine released by monocytes, macrophages, and non-immune cells in response to cellular injury and infection [32, 33]. Data in this study revealed that the lethal vvIBDV-infected chickens had high levels of IL-1*β* in all three immune organs (bursa, spleen, and thymus) and the blood, whereas the nonlethal nVarIBDV-infected chickens had low IL-1*β* levels. D279N mutation significantly reduced the IL-1*β* levels of rHLJ0504 in the immune organs and blood, whereas N279D mutation significantly increased the IL-*β* levels of rSHG19 in the bursa and thymus. Taken together, the D279N mutation of VP2 greatly reduced the viral replication and inflammatory factor levels in immune organs, such as the bursa, spleen, and thymus, and the viral titer and inflammatory factor levels in the blood, further reducing the systemic inflammatory storm and mitigating the damage on major immune organs and physiological functions of other organs, which may be the mechanism by which the D279N mutation reduced the lethality of vvIBDV from 70% to 0%. However, the mechanism by which residue 279 of VP2 affects IBDV pathogenicity requires further study.

The ability of nVarIBDV to escape the immune protection provided by the vvIBDV vaccine is responsible for its prevalence in immunized flocks. A neutralization test revealed a significant difference in the antigenicity between nVarIBDV and vvIBDV. The effect of residue 279 on IBDV antigenicity was also explored. D279N substitution reduced the neutralizing ability of HLJ0504 antiserum against the point-mutated virus by ∼15%, whereas the N279D substitution decreased the neutralizing ability of SHG19 antiserum by ∼14%. The data in both directions demonstrated that residue 279 of VP2 is a crucial amino acid for antigenic differences between the two prevalent IBDV strains. Therefore, capsid protein VP2 is the main protective antigen of IBDV. VP2 has an HVR on the outermost part of the viral particle and contains neutralizing antigenic epitopes responsible for binding to antibodies [34, 35]. Here, we found that the D279N mutation significantly reduced the affinity of the HLJ0504 antiserum for the VP2 mutant protein. D279N mutation interfered with the binding ability of the viral VP2 protein and antibody, decreasing the antigen–antibody affinity and neutralizing ability of the vvIBDV antiserum against nVarIBDV, which may be the molecular mechanism by which residue 279 causes the difference in antigenicity between the two dominant epidemic IBDV strains. Our neutralization test revealed that residue 279 was the key amino acid but not the only factor affecting viral antigenicity. We previously reported that residues 318 and 323 of P_HI_ in VP2 affect the antigenicity of IBDV [26]. However, other amino acids may affect the antigenicity of the virus or play synergistic roles with residue 279, which requires further study.

D279N substitution not only reduced viral lethality but also changed the antigenicity of vvIBDV. The phenotype changes caused by the introduction of N279D mutations into the SHG19 strain were significantly different compared to that of the parental nVarIBDV SHG19, although the changed features have not yet fully reached the level of vvIBDV HLJ0504. Two data lines confirmed that residue 279 of VP2 is the crucial molecular basis for the difference between nVarIBDV and vvIBDV. In addition, it is an understandable phenomenon that we could not see equivalent effects from mutations in different genome surroundings [10, 31, 36, 37]. Although individual amino acid substitution can significantly impact the biological characteristics of the virus, sometimes the degree of such impact is closely related to the genomic environment in which it is located. For IBDV, both segments contribute to viral replication efficiency and virulence, although segment A is the most important virulence factor (Escaffre et al., 2013) [27, 38–40]. Even for segment A, other amino acid differences in SHG19 compared with the HLJ0504 strain might influence the function of residue 279.

## 5. Conclusions

For the first time, we demonstrated that residue 279 of VP2 is the crucial molecular basis for the difference in pathogenicity between nVarIBDV and vvIBDV. We demonstrated that D279N substitution reduced the lethality of vvIBDV from 70% to 0%. The significant reduction in the viral load and inflammatory factor levels in the immune organs and blood of infected chickens may be important mechanisms for the decrease in the lethality of IBDV. Moreover, residue 279 was further identified as the important molecular basis for the antigenic differences between nVarIBDV and vvIBDV. D279N substitution reduced the neutralizing ability of the vvIBDV antiserum against nVarIBDV by affecting the binding ability of the antibody and antigen. Our results indicate that nVarIBDV has an infection-transmission strategy that facilitates survival by hiding its pathogenicity and escaping antiserum neutralization. This study has significant implications for the systemic cognition of viral genetic evolution and pathogenesis and provides new ideas for the comprehensive prevention and control of IBDV.

## Figures and Tables

**Table 1:** The VP2 gene information of the reference IBDV strains.

Phenotype	Reference strain	Origin	GenBank number
nVarIBDV	SHG19	China	MN393076
	19D38	Korea	MT550875
	UPM14322019	Malaysia	MT505348
	Kagoshima MO 2B-28	Japan	MN171482

vvIBDV	HLJ0504	China	GQ451330
	HK46	China	AF092943
	D6948	Netherlands	AF240686
	UK661	UK	NC-004178

**Table 2:** Primers or probes.

Primers	Sequence (5′-3′)	Purpose
pCA-EcoR1-F	TCTCATCATTTTGGCAAAGAATTC	Point mutation
pCA-Bgl2-R	ATTTTTGGCAGAGGGAAAAAGATCT	
HLJVP2-D279N-F	CACCAGAGCTGTGGCCGCAAACAATGGGCTAACGGCCGGCAC	
HLJVP2-D279N-R	GTGCCGGCCGTTAGCCCATTGTTTGCGGCCACAGCTCTGGTG	
SHG19VP2-N279D-F	AGAGCTGTAGCTGCAGACAATGGGCTGACGG	
SHG19VP2-N279D-R	CCGTCAGCCCATTGTCTGCAGCTACAGCTCT	
PCA-HLJVP2-F	TCTCATCATTTTGGCAAAGAATTCGCCACCATGACAAACCTGCAAGATC	
PCA-HLJVP2-R	TTGGCAGAGGGAAAAAGATCTTTAAGCGTAATCTGGAACATCGTATGGGTATGCTCCTGCAATCTTCAG	
IBDV-Probe	FAM-CGGCGTCCATTCCGGACGAC-BHQ-1	RT-qPCR for IBDV
VP5-F	GAGCCTTCTGATGCCAACAAC	
VP5-R	CAAATTGTAGGTCGAGGTCTCTGA	
28S-Probe	FAM-AGGACCGCTACGGACCTCCACCA-TAMRA	RT-qPCR for 28s rRNA
28S-F	GGCGAAGCCAGAGGAAACT	
28S-R	GACGACCGATTTGCACGTC	
IL-1*β*-F	CCGAGGAGCAGGGACTTT	RT-qPCR for IL-1*β*
IL-1*β*-R	AGGACTGTGAGCGGGTGTAG	
*β*-actin-F	TCCACCGCAAATGCTTCTAAAC	RT-qPCR for *β*-actin
*β*-actin-R	CTGCTGACACCTTCACCATTCC	

## Data Availability

The data that support the findings of this study are available from the corresponding author upon reasonable request.
